# Dynamic Succession of the Early-Life Gut Microbiota and the Regulatory Role of Human Milk Oligosaccharides

**DOI:** 10.3390/nu18162621

**Published:** 2026-08-11

**Authors:** Jia Yin, Zhixu Wang, Ye Ding

**Affiliations:** 1 Department of Maternal, Child and Adolescent Health, School of Public Health, Nanjing Medical University, Nanjing 211166, China; juju@stu.njmu.edu.cn (J.Y.); zhixu_wang@163.com (Z.W.); 2 The Institute of Nutrition and Food Science, Nanjing Medical University, Nanjing 211166, China

**Keywords:** early-life gut microbiota, human milk oligosaccharides, infant feeding, microbial succession

## Abstract

Early-life nutrition and the gut microbiota interact to shape host health programming. During early-life development, spanning the neonatal period of initial gut colonization through infancy to toddlerhood, the gut microbiota undergoes dynamic succession driven by feeding patterns, host genetic background, environmental exposures, and other intrinsic and extrinsic factors, among which early feeding practices play a particularly prominent role. Among these factors, human milk oligosaccharides (HMOs), key bioactive components of human milk, contribute to the shaping of infant gut microbial ecology by selectively supporting HMO-utilizing bacteria, influencing microbial metabolism and cross-feeding, and exhibiting structure- and context-dependent effects on pathogen–host interactions, intestinal barrier function, and immune responses. This narrative review summarizes the succession patterns of the gut microbiota in early life and the core regulatory mechanisms by which HMOs shape infant gut microbial ecology, aiming to provide a conceptual basis for understanding the potential long-term health implications of early nutritional interventions.

## 1. Introduction

Early life represents a critical window for the establishment and evolution of the human gut microbiota, with sustained and far-reaching effects on health across the life course [1]. These effects are not confined to growth, metabolic development, and immune function during childhood and adolescence, but may extend into adulthood, where alterations in the gut microbiota have been associated with the risk of multiple chronic non-communicable diseases [2,3]. Gut microbiota maturation is a non-random process. In early life in particular, as infant dietary patterns change in a stage-specific manner, distinct signature species and dynamic networks of positive and negative interactions among key microbial taxa can be identified across different developmental windows [4,5]. During the first 1000 days of life, the microbiota undergoes a maturation process from low diversity and high variability to relatively high diversity and stability. Taking breastfed infants as an example, a relatively well-defined pattern of microbial succession can be observed [6]: the first few weeks are characterized by colonization by facultative anaerobes, followed by a period during the first 6 months of life in which bifidobacteria typically predominate; with the introduction of complementary foods, major human gut bacterial phyla, including Bacteroidota and Firmicutes, gradually form more diverse microbial communities. Characterizing these stage-specific trajectories is therefore essential for identifying developmental windows during which early dietary exposures may guide microbiota maturation and inform microbiota-centered nutritional strategies.

Extensive studies have shown that maternal and infant diet, mode of delivery, environment, lifestyle, and other factors are key determinants of microbial succession and future health outcomes [7,8,9]. In the context of breastfeeding, the strains acquired by the infant are further shaped by maternal diet, ethnicity, cultural practices, and household environment. For instance, a dietary intervention trial demonstrated that reducing fat and sugar intake while increasing fiber for two weeks significantly altered the human milk microbiota, including an increase in *Cutibacterium acnes* and a decrease in *Haemophilus parainfluenzae* [10]. Likewise, milk microbiota profiles differ by ethnicity, as shown by distinct compositions among Asian, Māori, and European women in New Zealand [11]. Additionally, birth setting and breastfeeding practices may further shape the timing, retention, and functional diversity of vertically transmitted microbial strains [9]. Together, these findings indicate that the breastfed infant gut microbiota reflects an interplay of maternal, environmental, and cultural factors rather than a uniform state.

Among numerous modifiable factors, early-life nutrition—particularly feeding mode—is a particularly prominent and critical driver of microbiota development and serves as the central focus of this review [12]. Human milk is a highly evolved bioactive system and the natural food best suited to meet infant nutritional requirements. It not only provides essential nutrients but also contains a wide range of bioactive molecules. Among these, human milk oligosaccharides (HMOs) are the third most abundant solid component after lactose and lipids. Along with other bioactive factors, including lactoferrin and immunoglobulins, HMOs contribute to the regulation of the infant gut microbiota and immune function [13,14]. HMOs cannot be directly digested by the infant gastrointestinal tract; however, they can serve as selective substrates for bacteria, promote the growth of beneficial microbes such as bifidobacteria, and exert multiple important physiological functions [15]. Previous work has described the maturation of the infant gut microbiota and the role of early-life diet in this process, with human milk oligosaccharides (HMOs) recognized as important bioactive components of human milk. However, dietary regulation is often discussed in broad terms, particularly through the contrast between breastfeeding and formula feeding, whereas real-world feeding contexts such as donor human milk and mixed feeding remain less consistently characterized [16]. This review therefore first outlines the stage-specific succession of the gut microbiota from neonatal colonization to toddlerhood, with attention to dietary influences across early life. It then focuses on the regulatory role of HMOs in this developmental process, linking HMO structural specificity and strain-dependent bifidobacterial utilization to metabolite production, cross-feeding networks, colonization resistance, intestinal barrier support, and immune regulation.

## 2. Methods

### 2.1. Literature Search Strategy

This narrative review was informed by a structured literature search of PubMed and the Web of Science Core Collection from database inception through June 2026. No publication-date restrictions were applied, and only articles published in English were considered. In PubMed, free-text terms were searched in the Title/Abstract fields, whereas corresponding terms were searched in the Topic field of the Web of Science Core Collection. These terms covered the principal themes of the review, including “early-life gut microbiota”, “infant gut microbiome”, “microbial succession”, “breastfeeding”, “donor human milk”, “formula feeding”, “mixed feeding”, “complementary feeding”, “human milk oligosaccharides”, “HMOs”, “*Bifidobacterium*”, “HMO utilization”, “microbial metabolites”, “cross-feeding”, “colonization resistance”, “intestinal barrier”, and “immune regulation”. Relevant synonyms and specific HMO structures, including 2′-fucosyllactose, lacto-*N*-tetraose, lacto-*N*-neotetraose, and sialyllactose, were incorporated where appropriate. Search terms were combined using the Boolean operators AND and OR. The reference lists of relevant original studies and review articles were also screened manually to identify additional publications pertinent to the scope of the review.

### 2.2. Inclusion Criteria

Primary studies were considered eligible when they addressed at least one of the following topics: the establishment and stage-specific succession of the gut microbiota from the neonatal period to toddlerhood; the influence of birth-related exposures or infant feeding practices on microbial composition and function; the structure-specific or strain-dependent utilization of HMOs; or the effects of HMOs on microbial metabolism, cross-feeding, pathogen–host interactions, intestinal barrier function, or immune regulation. Eligible evidence included prospective and retrospective human observational studies, randomized and non-randomized feeding interventions, preclinical animal studies, and mechanistic in vitro experiments.

Systematic and narrative reviews, consensus statements, and authoritative guidelines were consulted to provide background context, summarize established concepts and broader evidence patterns, and identify relevant primary studies. When specific experimental, microbiological, or clinical findings were discussed, priority was given to the corresponding original studies.

### 2.3. Exclusion Criteria

Records were initially assessed on the basis of titles and abstracts, followed by full-text evaluation of articles considered relevant to the objectives of the review. Studies were excluded when they did not directly address early-life gut microbial development, infant feeding, or HMO-related biological mechanisms; provided insufficient methodological information to permit meaningful interpretation of the findings; consisted solely of conference abstracts, commentaries, editorials, or other non-peer-reviewed material; or substantially duplicated evidence already represented by a more informative or methodologically robust study.

### 2.4. Evidence Interpretation

Because this was a narrative rather than a systematic review, no formal risk-of-bias assessment or quantitative evidence-grading framework was applied. Nevertheless, evidence interpretation was guided by a structured qualitative synthesis framework incorporating the following dimensions: study design type, study population or experimental model, sample size, characterization of the feeding patterns or HMO exposure, microbiome or laboratory methodology, appropriateness of the comparator, and the clinical relevance of the reported outcomes. In the narrative synthesis, in vitro, animal, observational human, and human intervention studies were treated as distinct categories of evidence, and mechanistic or preclinical findings were not considered equivalent to clinical outcomes. The evidence was organized according to the major themes of the review, while differences in study design, methodology, and translational relevance were considered when interpreting apparently consistent or divergent findings.

## 3. Stage-Specific Succession of the Early-Life Gut Microbiota and Its Dietary Regulation

### 3.1. Critical Windows in Early-Life Gut Microbiota Development

#### 3.1.1. The Pre-Complementary-Feeding Period: Initial Colonization and Early Microbial Succession

The gut microbiome in early postnatal life is highly dynamic, and its establishment during this period is generally sensitive to perinatal factors [17,18]. The study by Ferretti et al. demonstrated that mothers share microbes with their infants through multiple routes, including feces, human milk, vaginal contact, and skin, suggesting that mother–infant microbial sharing is an important source for the early establishment of the neonatal gut microbiota [19]. In addition to maternal sources, close household contacts and the birth environment may also contribute to early microbial community assembly. Strain-resolved studies have identified fathers as a stable source of infant gut microbial strains [20] and have shown that birth setting can influence mother-to-infant transmission dynamics [9]. However, the relative contribution and long-term persistence of non-maternal strains remain incompletely understood and may vary across settings. Among these early exposures, birth represents a major microbial encounter. Accordingly, mode of delivery is considered a key factor shaping the early seeding and development of the neonatal gut microbiota. Previous studies have confirmed significant differences between vaginally delivered and cesarean-section-born infants in gut microbiota composition, diversity, and the transmission rate of maternal strains [21].

Vaginal delivery provides neonates with direct and extensive exposure to microbes from the maternal birth canal and perianal region. A large Swedish cohort study found that, among vaginally delivered infants, up to 72% of gut bacteria within 2–5 days after birth could be traced to the maternal gut, including *Escherichia/Shigella*, *Bifidobacterium longum*, *Enterococcus faecalis*, *Bacteroides fragilis*, *Bacteroides thetaiotaomicron*, and *Bilophila wadsworthia*, reflecting a high degree of mother–infant strain concordance [4]. This transmission pattern enables the early infant gut to rapidly establish beneficial microbial niches centered on bifidobacteria and *Bacteroides*. The persistence of maternally derived strains for a period after initial colonization suggests that the transmitted fraction of the maternal gut microbiota may have protective functions by preventing the influx of environmental conspecific strains, which may carry a higher risk of unfavorable traits [4]. However, it remains unclear why maternal strains are more stable than non-maternal conspecific strains and how long maternal strains persist.

In contrast, because cesarean-section-born infants lack direct microbial exposure during passage through the birth canal, both the sources of their early colonizers and the patterns of community assembly are markedly altered. Only approximately 40% of the gut microbiota in these infants originates from the maternal gut [4]. In infants delivered by cesarean section, disrupted vertical transmission of maternal commensals is accompanied by increased colonization by hospital-associated opportunistic pathogens, particularly *Enterococcus*, *Enterobacter*, and *Klebsiella* species [22]. More recent metagenomic evidence further indicates that cesarean birth is associated with an *Enterococcus faecalis*-dominated neonatal gut community state, which frequently co-occurs with environment- and skin-associated *Streptococcus* and *Staphylococcus* spp., as well as healthcare-associated opportunistic pathogens including *Enterococcus*, *Klebsiella*, *Enterobacter* spp. and *Clostridium perfringens* [23]. Thus, the early microbiota of cesarean-section-born infants is characterized by significantly delayed or reduced colonization by key beneficial bacteria of maternal gut origin, such as *Bacteroides*, a potentially higher relative abundance of opportunistic pathogens, and lower overall similarity to the maternal gut microbiota.

Nevertheless, the impact of delivery mode on the microbiota is not necessarily permanent; its duration and biological significance may be jointly modulated by subsequent feeding mode, antibiotic exposure, and environmental exposures. For example, in predominantly breastfed infants, the selective nutritional substrates and immunologically active components provided by human milk may partially buffer delivery-related microbiota perturbations [24].

At birth, the infant gut still contains a certain amount of oxygen and therefore represents a relatively aerobic environment. The earliest colonizers are mostly facultative anaerobes, such as *Escherichia* and *Enterococcus* [17]. These bacteria can consume residual oxygen in the gut and gradually reduce the local redox potential, thereby driving the transition of the intestinal environment from aerobic to anaerobic [25]. As oxygen levels decline, obligate anaerobes begin to proliferate, including members of the class Clostridia within Firmicutes, the genus *Bacteroides*, and the genus *Bifidobacterium*, a key component of the infant gut microbiota. Within approximately 2 weeks after birth, the diversity and richness of the neonatal gut microbiota gradually increase, although the overall structure remains dominated by a small number of predominant microbial groups. The core microbiota of the early infant gut mainly includes bifidobacteria, *Veillonella*, *Streptococcus*, *Citrobacter*, *Escherichia*, as well as *Bacteroides* and clostridial groups [26]. Among these, bifidobacteria are particularly important in early life, with their relative abundance reaching as high as 37% of the infant gut microbiota [26].

These early colonization patterns should be interpreted within the dietary context of the first months of life, during which human milk, infant formula, or a combination of both usually constitutes the predominant source of nutrition before complementary foods are introduced. Compared with the post-weaning dietary environment, this period is characterized by a relatively restricted substrate spectrum, mainly composed of milk-derived carbohydrates, proteins, lipids, and bioactive compounds. Longitudinal studies have shown that infant gut microbiota development during the first weeks to months follows staged successional patterns, with early communities enriched in facultative anaerobes and birth- or skin-associated taxa gradually giving way to communities in which Bifidobacteriaceae often become prominent [27,28]. However, this pattern is not uniform across infants. Cross-lifestyle and cross-population metagenomic studies indicate that early microbial assembly reflects both conserved developmental trajectories and population-specific feeding, environmental, and lifestyle contexts; for example, a *Bifidobacterium*–*Streptococcus* configuration is common during the first 6 months, whereas increasing age is generally associated with a decline in *Bifidobacterium* and expansion of taxa such as *Faecalibacterium prausnitzii* and members of Lachnospiraceae [29,30]. Therefore, the pre-complementary-feeding period is best viewed as an early milk-feeding stage in which microbial succession is shaped by the interaction between initial seeding, host developmental conditions, and milk-related nutritional substrates, rather than as a uniform breastfeeding-specific state. The differential effects of breastfeeding, donor human milk, formula feeding, and mixed feeding on this trajectory are discussed separately in Section 3.2.

#### 3.1.2. The Complementary-Feeding Transition and Toddlerhood: Dietary Diversification and Microbiota Maturation

Following this early milk-feeding stage, the introduction of complementary foods represents a second critical window of ecological transition in infant gut microbiota development. However, compared with the relatively well-characterized effects of human milk and infant formula on early microbial colonization, evidence remains more limited regarding how complementary feeding, weaning, and toddler diets jointly shape gut microbial trajectories. In real-world feeding practices, complementary feeding is often intertwined with reduced human milk intake, increased formula intake, and the progression of weaning, making it difficult to attribute microbiota changes during this period to any single feeding event. In infant and young child feeding, complementary foods are generally recommended to be introduced at around 6 months of age [31]. As fruits, vegetables, cereals, porridges, bread, meat, dairy products, legumes, and other foods are gradually incorporated into the infant diet, the intestinal substrate supply shifts from predominantly milk-derived carbohydrates, such as human milk oligosaccharides (HMOs) and lactose, to a more diverse nutritional environment that includes dietary fiber, complex carbohydrates, proteins, and peptides. This increase in dietary complexity is associated with a transition of the gut microbiota from an early, milk-adapted, low-diversity community toward a toddler-like microbiota with enhanced capacity for complex substrate degradation. During this stage, the abundance of HMO-degrading *Bifidobacterium* species decreases, the gut microbiota shows a marked increase in α-diversity, and members of the bacterial families Bacteroidaceae, Lachnospiraceae, and Ruminococcaceae increase in abundance [32]. Dietary fiber is a preferred energy source for gut microbes, whereas incompletely digested proteins and peptides constitute another important class of substrates [33]. Therefore, changes in the sources of fiber and protein in complementary foods are likely to be important drivers of structural and functional maturation of the gut microbiota during this stage [32].

The compositional shift in the microbiota is accompanied by maturation of microbial metabolic functions. Fermentation of dietary fiber produces short-chain fatty acids (SCFAs), mainly including acetate, propionate, and butyrate [34]. In the early infant gut, metabolites such as acetate and lactate are relatively common, whereas butyrate and propionate are generally present at low levels; these metabolites then gradually increase with the introduction of complementary foods and advancing age [35]. Meanwhile, protein fermentation-related metabolites, such as branched-chain fatty acids (BCFAs), are barely detectable during the breastfeeding period but also increase progressively with age [35]. These dynamic changes in SCFAs and BCFAs usually overlap temporally with the introduction of solid foods and the cessation of breastfeeding, reflecting the succession of the infant gut microbiota from a milk-adapted state toward a complex diet-adapted state.

**Figure 1:**
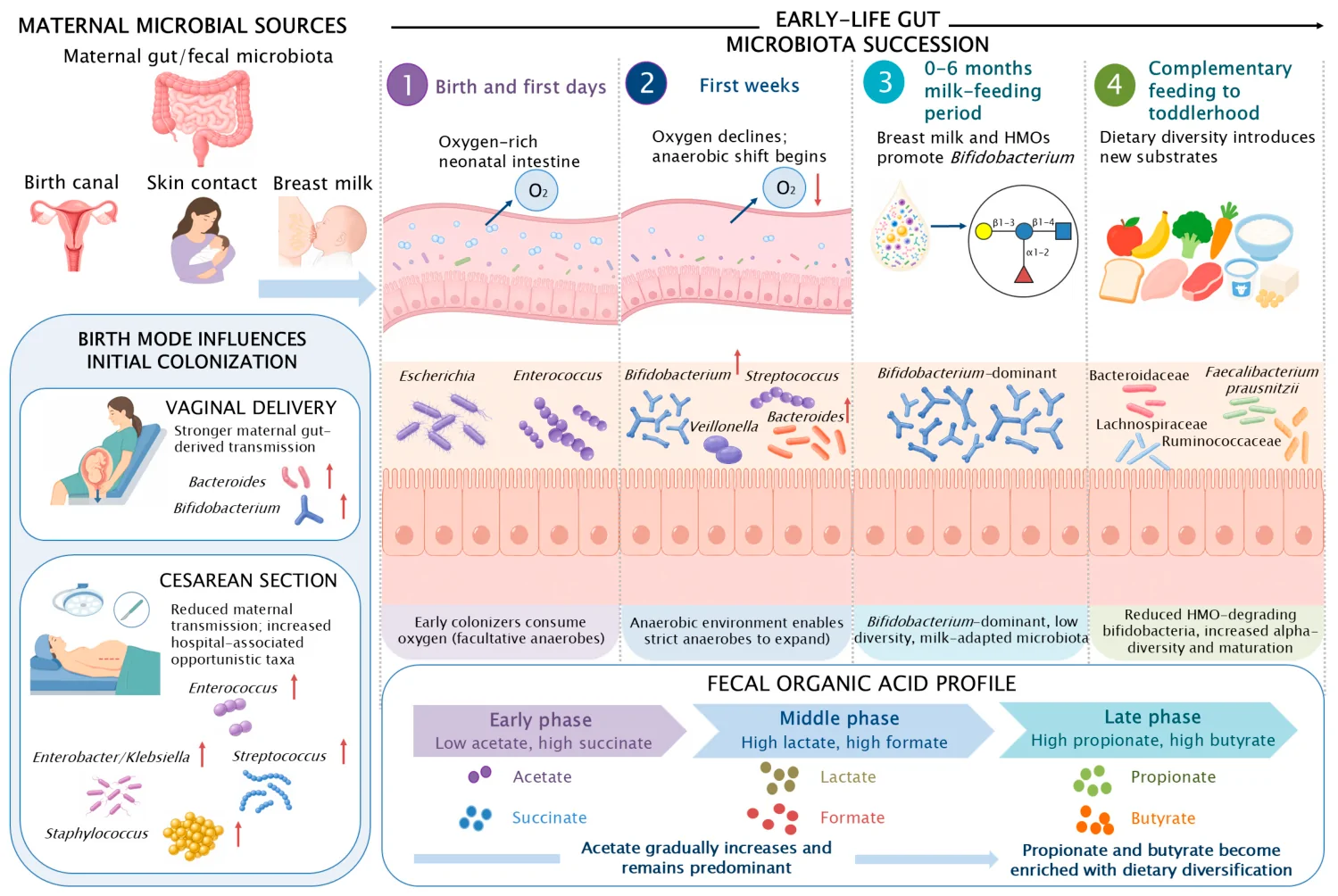
Maternal seeding, birth mode, and diet-driven succession of the early-life gut microbiota and associated fecal organic acid profiles. The fecal organic acid profiles follow a generalized three-phase longitudinal model; microbial succession and metabolite patterns may vary among infants. Upward and downward arrows indicate relative increases and decreases, respectively.

To provide an integrated overview of the early-life microbial trajectory discussed above, Figure 1 summarizes the major stage-related changes in gut microbiota composition and metabolite profiles, with particular emphasis on the early influence of birth mode and the subsequent dietary transition from milk feeding to complementary feeding.

### 3.2. Effects of Infant Feeding Mode on Gut Microbial Colonization and Succession

#### 3.2.1. Breastfeeding: Promoting Bifidobacterial Dominance and the Establishment of a Milk-Adapted Microbiota

According to joint guidelines from the World Health Organization and pediatric societies, breastfeeding should be initiated within the “golden first hour” after birth, exclusive breastfeeding should be maintained for 6 months, and breastfeeding should continue up to 2 years of age [36]. Human milk is not only a biologically complex and functionally diverse fluid but also a material foundation that supports appropriate microbial colonization and growth in the infant gut [37]. Breastfeeding has been consistently associated with substantial short- and long-term health benefits in infants. A recent review summarized evidence associating breastfeeding with lower risks of respiratory, gastrointestinal, and ear infections and septicemia, as well as, particularly among preterm infants, necrotizing enterocolitis and late-onset sepsis. Longer-term associations have also been reported for obesity, type 1 diabetes, and childhood leukemia [38]. These health benefits are likely multifactorial but may be partly attributable to the early establishment of a balanced and resilient microbiota, which is thought to contribute to the regulation of metabolism, immune responses, intestinal barrier function, and homeostasis [12]. Current evidence indicates that the essential macronutrients and micronutrients supplied by human milk support optimal microbial growth, development, and functional maturation in the infant gut [39,40,41]. In addition, milk-derived microbes can be transferred to the infant oral cavity and gut during breastfeeding, constituting a unique source of early-life microorganisms [42].

Breastfed infants exhibit a distinctive gut microbial profile, characterized mainly by lower overall microbial diversity and richness, together with a higher relative abundance of beneficial bacteria such as bifidobacteria. A meta-analysis of seven infant gut microbiome studies, integrating 1825 fecal samples from 684 infants, showed that, compared with non-exclusively breastfed infants, exclusively breastfed infants consistently had lower gut bacterial diversity, microbiota maturity, relative abundances of Bacteroidota and Firmicutes, and predicted microbial pathways related to carbohydrate metabolism; these differences could persist beyond 6 months of age [43]. Another large multicenter study longitudinally followed 903 children aged 3–46 months, collected fecal samples monthly, and analyzed gut microbial composition using 16S rRNA gene sequencing [44]. The results showed that breastfed infants had higher fecal relative abundances of *Bifidobacterium*, particularly *Bifidobacterium breve* and *Bifidobacterium bifidum*. Notably, breastfeeding not only shapes a distinctive gut microbial community in infants but also helps maintain its ecological homeostasis. Diarrhea is one of the most common diseases in infancy and usually induces gut microbiota disruption for a certain period [45,46]. One study found that diarrhea was closely associated with loss of microbial diversity, reduced microbiota age, decreased relative abundance of Bifidobacteriaceae, and increased relative abundance of Streptococcaceae [43]. However, these phenomena were rarely observed in infants who had received exclusive breastfeeding for more than 2 months or in infants who were still being breastfed at the time of diarrhea. These findings suggest that longer-duration breastfeeding may help enhance the stability and resilience of the infant gut microbiota, thereby playing an important role in maintaining intestinal health in early life.

In recent years, research on donor human milk (DHM) has gradually increased, focusing mainly on differences in nutritional and bioactive components between DHM and parent’s own milk (POM), as well as differences in feeding outcomes among DHM, POM, and formula [47,48,49]. When POM is unavailable or insufficient, DHM is considered the next-best option, particularly for high-risk infants such as preterm infants and very-low-birth-weight infants [50]. Although guidelines and policies for human milk bank management, quality control, safety regulation, and ethical standards vary across countries and regions, DHM generally undergoes screening, processing, and pasteurization to reduce the risk of bacterial and viral contamination and to ensure feeding safety for vulnerable preterm neonates [50]. While pasteurization preserves most nutrients, it can also markedly alter milk composition and reduce its bioactivity to some extent [51,52]. These differences in nutritional and functional components may be further reflected in alterations in the structure of the infant gut microbiota. A systematic review summarized the available evidence and suggested that, compared with infants receiving POM, DHM-fed infants had lower gut microbial α-diversity indices, higher abundances of Staphylococcaceae and Clostridiaceae, and relatively lower abundances of Bacteroidota and *Bifidobacterium* [53]. At present, studies on the effects of DHM on the infant gut microbiota remain limited, and existing studies show substantial heterogeneity in study populations, feeding practices, DHM processing procedures, detection technologies, and follow-up durations. More rigorously designed prospective cohort studies and multicenter studies are needed to further clarify the effects of DHM feeding on infant gut microbial colonization, microbiota maturation, and long-term health outcomes, and to explore the potential effects of different processing methods on the bioactive components of DHM and their microecological functions.

#### 3.2.2. Formula Feeding: Increased Microbial Diversity and Earlier Emergence of Adult-like Features

Infant formula differs from human milk in nutrient composition, bioactive factors, and ecological regulatory functions, making it difficult to fully reproduce the integrated effects of breastfeeding on infant growth, development, and gut microbial establishment. This is particularly evident in the characteristics of the infant gut microbiota. Compared with breastfed infants, formula-fed infants harbor a more diverse gut microbiota that includes members of the family Enterobacteriaceae and genera such as *Streptococcus*, *Bacteroides*, *Bifidobacterium*, *Clostridium*, and *Atopobium*; these infants generally exhibit higher gut microbial diversity and a more rapid trend toward microbiota maturation, whereas the relative abundance of typical breastfeeding-associated beneficial bacteria, such as bifidobacteria, is markedly reduced [54]. Meanwhile, the study by Pärnänen et al. provided innovative evidence that formula feeding may alter the early gut microbiota, promoting the enrichment of facultative anaerobes carrying antibiotic resistance genes (ARGs) and thereby increasing the risk of expansion of potential pathogens [55].

With advances in food processing and nutritional fortification technologies, the composition of modern infant formula has been continuously optimized. Several randomized controlled trials have shown that fortified formulas outperform standard formulas in some nutritional outcomes and, to a certain extent, approach the effects observed with breastfeeding [56,57]. In a double-blind randomized controlled trial, Estorninos et al. demonstrated that consumption of formula supplemented with bovine milk-derived oligosaccharides during the first 4 months after birth could shift the overall microbiota composition toward a profile more similar to that of breastfed infants and induce a significant bifidogenic effect [58]. In a randomized controlled trial, Zuffa et al. further suggested that formula containing large phospholipid-coated lipid droplets was associated with lower abundances of selected opportunistic taxa and enrichment of selected butyrate-associated taxa, with multi-omic trajectories closer to those of the breastfed reference group [59]. However, different formula fortification strategies vary substantially in their components, dosages, and feeding durations, resulting in a certain degree of heterogeneity across studies. Moreover, existing research has mainly focused on short-term effects during the first 4–6 months after birth, whereas systematic evidence regarding long-term health outcomes and immunomodulatory effects remains limited. Future studies should further explore strategies for optimizing combinations of fortified nutrients in infant formula, evaluate their effects on the long-term development of the infant gut microbiota, immune function, and health outcomes, and integrate strain-level and functional omics approaches to provide theoretical and practical support for more precise simulation of the bioactivity of human milk by infant formula.

#### 3.2.3. Mixed Feeding: Interactions Between the Protective Effects of Human Milk and the Effects of Formula

Because of limitations related to maternal milk supply, work or life stress, postpartum health status, social support, and other factors, many families are unable to maintain exclusive breastfeeding; therefore, mixed feeding is a common feeding practice. At present, studies examining how mixed feeding affects the composition and metabolic status of the infant gut microbiome remain relatively limited, and their conclusions are inconsistent. Overall, the relatively clear finding is that formula exposure during mixed feeding is associated with microbial and metabolic profiles that differ from those observed in exclusively breastfed infants. Based on the STRONG Kids 2 birth cohort, Wang et al. used metagenomic sequencing and untargeted metabolomics to compare fecal microbiome and metabolome profiles among exclusively breastfed, exclusively formula-fed, and mixed-fed infants at 6 weeks of age [60]. The results showed that α-diversity and β-diversity in mixed-fed infants differed significantly from those in exclusively breastfed infants but were similar to those in formula-fed infants, with microbial functional pathways showing a comparable pattern. This study proposed that, even with partial human milk intake, formula exposure may rapidly alter gut microbial ecology and metabolic function in early life, causing mixed-fed infants to exhibit a microbial–metabolic phenotype more similar to that of formula-fed infants [60]. Myers et al. further confirmed in two European birth cohorts that formula supplementation can exert detectable effects on infant metabolism and gut microbial ecology even during the breastfeeding period. This study also suggested that mixed feeding is not simply an intermediate state between exclusive breastfeeding and exclusive formula feeding; instead, it may lead to varying degrees of deviation depending on the amount of formula intake, timing of exposure, and formula composition [61]. However, because formula composition was not consistently characterized across the available studies, the contributions of individual formula components to the observed microbial and metabolic differences remain unclear.

Notably, human milk intake varies substantially among mixed-fed infants, and the proportion of human milk and formula intake often changes dynamically over time [62], making assessment of its microbial effects more complex and potentially contributing to differences among existing studies. Classification based solely on whether an infant is mixed-fed may obscure dose–response relationships arising from different levels of human milk intake. Currently, the deuterium oxide dose-to-mother technique and 24 h test-weighing are commonly used to quantify infant human milk intake in research [63]. The deuterium oxide dilution method estimates the amount of human milk consumed by the infant over a defined period by tracking changes in deuterium enrichment in maternal and infant body fluids; because it can be performed under relatively natural feeding conditions, it is well suited for population-based and cohort studies [64]. Test-weighing is straightforward to perform but is easily affected by weighing precision, feeding frequency, and operational procedures [65,66]. However, after identifying 167 studies on human milk intake, Rios-Leyvraz et al. noted that the deuterium dilution method often yields higher estimates than test-weighing [63]. In recent years, real-time monitoring technologies based on breast-worn wireless sensor devices have also been developed; these devices estimate milk release by detecting local physiological signal changes in the breast during feeding [67]. This provides a new technical approach for dynamically assessing human milk intake in home settings, although its accuracy, target populations, and scalability in large cohorts still require further validation.

Representative human studies addressing early-life gut microbiota succession and exposures related to infant feeding, human milk, or HMOs are summarized in Table 1.

## 4. Core Mechanisms by Which Human Milk Oligosaccharides Regulate the Gut Microbiota

### 4.1. Selective Microbiota-Shaping Effects of HMOs as Natural Prebiotics

#### 4.1.1. Specific Promotion of the Proliferation of Bifidobacteria and Other Beneficial Bacteria

Among the diverse bioactive factors through which early feeding practices shape infant gut microbial succession, HMOs represent one of the best-characterized milk-derived ecological selectors. Structurally, HMOs are commonly classified into three major groups: neutral fucosylated HMOs, acidic sialylated HMOs, and neutral non-fucosylated HMOs, reflecting differences in fucosylation and sialylation patterns [68]. Human milk HMO composition has been associated with variation in the infant gut microbiota [70]. Because the infant digestive system lacks the enzymes required for HMO catabolism, most HMOs are resistant to gastrointestinal enzymatic degradation and can reach the lower intestine [15], where they become available as substrates for selected infant-associated microbes. Ex vivo experimental evidence further indicates that HMOs can be utilized by *Bifidobacterium longum* subsp. *infantis* and can contribute to microbial metabolite production [71]. This microbiota-directed property provides a mechanistic basis for linking human milk glycan composition to strain-specific microbial metabolism and the preferential expansion of infant-adapted bifidobacteria [72]. In recent years, studies focusing on HMO structural specificity, strain-specific metabolic preferences, and interindividual variation have provided new scientific evidence for elucidating the mechanisms underlying HMO–microbiota interactions. However, these mechanistic findings primarily explain microbial substrate utilization and metabolic responses and do not by themselves establish stable bacterial enrichment or clinical benefits in breastfed infants.

Through their complex and specific molecular structures, HMOs can selectively support the preferential expansion of infant-adapted bifidobacteria that carry the corresponding transport systems and glycoside hydrolase repertoires. Mechanistic studies suggest that HMO utilization may provide selected bifidobacterial strains with an ecological advantage through efficient substrate acquisition and metabolism, thereby potentially contributing to bifidobacteria-enriched microbial communities [73]. Bifidobacteria were first discovered by Tissier in 1899 from fecal samples of breastfed infants and are defined as Gram-positive anaerobes that generally have a high genomic G+C content [54]. The gut microbiota of healthy breastfed infants is typically dominated by “infant-type” bifidobacteria, including *B*. *longum* subsp. *infantis*, *Bifidobacterium longum* subsp. *longum*, *B. bifidum*, and *B. breve* [42,74]. Genomic studies have identified an enrichment of genes involved in HMO uptake, transport, and degradation in these strains, including genes encoding multiple sugar transporters and glycoside hydrolases [75,76,77]. These genomic features are consistent with the capacity to utilize selected HMOs and may provide an ecological advantage in an HMO-rich environment.

*Bifidobacterium longum* possesses an extensive HMO metabolic system, and its integrated transporter network, glycoside hydrolase machinery, and regulatory factors enable efficient utilization of diverse HMOs. Among them, *B. longum* subsp. *infantis* is regarded as a representative taxon with the strongest capacity for HMO utilization. Studies have shown that this bacterium can directly transport multiple HMOs into the cell through ATP-binding cassette (ABC) transport systems and subsequently degrade them using fucosidases, β-galactosidases, *N*-acetylhexosaminidases, sialidases, and other enzymes. Therefore, it has a strong capacity to utilize neutral, fucosylated, and some sialylated HMOs [75]. However, *B. longum* subsp. *infantis* and *B. longum* subsp. *longum* differ substantially in their ability and strategies for HMO utilization. Although they belong to the same species, *B. longum* subsp. *longum* lacks certain relevant glycoside hydrolases, which limits its capacity to efficiently utilize some HMOs [78]. The HMO utilization capacity of *B. longum* subsp. *longum* is clearly strain-specific and relatively limited, usually restricted to LNT and LNB; only a few strains can utilize other HMOs, such as 2′-FL, 3-FL, LNnT, and LNFP I/II/III [78,79,80]. *B. bifidum* mainly utilizes HMOs through an extracellular strategy. It relies more heavily on extracellular glycoside hydrolases, first degrading HMOs outside the cell and then absorbing the resulting small-molecule substrates [81]. *B. breve* has a relatively limited capacity for direct utilization of complex HMOs, with marked differences among strains, and more commonly utilizes selected neutral oligosaccharides or metabolic intermediates released by other bacteria [82]. Collectively, these findings establish species- and strain-specific mechanisms of HMO utilization under experimental conditions. Whether these metabolic differences translate into stable colonization advantages or clinically relevant outcomes in infants requires confirmation from longitudinal human studies and intervention trials.

#### 4.1.2. Generation of HMO-Related Metabolites

Bifidobacteria equipped with HMO transport systems and glycoside hydrolase repertoires can progressively degrade complex HMOs into monosaccharides and oligosaccharide fragments, which are subsequently fermented into organic acid metabolites. These metabolites reflect microbial HMO utilization and may contribute to modulation of the intestinal chemical environment and host responses [83,84]. Bifidobacteria mainly convert carbohydrate substrates derived from HMO degradation into end products such as acetate, lactate, formate, and small amounts of ethanol through the fructose-6-phosphate phosphoketolase pathway [85]. In vitro studies have identified acetate and lactate as major fermentation products generated during HMO utilization by infant-associated bifidobacteria [86,87]. By lowering environmental pH, the accumulation of these organic acids can favor acid-tolerant commensals and restrict the growth of selected pH-sensitive microorganisms under experimental conditions [88].

Different HMO structures can generate distinct metabolite profiles under experimental conditions. An in vitro batch-fermentation study using seven *B. longum*-dominant infant fecal inocula investigated microbial and metabolic responses to six individual HMOs [89]. In microbiota dominated by *B. longum*, all six HMOs produced similar levels of acetate, whereas neutral HMOs such as 2′-FL, 3-FL, LNT, and LNnT produced more lactate than sialylated HMOs such as 3′-SL and 6′-SL. This finding suggests that HMOs do not function as a single, uniform prebiotic; rather, differences in fucosylation, sialylation, and core chain type can influence microbial utilization and metabolite production in vitro. In a pure-culture study of *B. longum* subsp. *infantis* Bi-26, growth on 2′-FL was accompanied by the production of formate, acetate, lactate, and 1,2-propanediol and by the cleavage of fucose from 2′-FL [90]. Lactate and succinate can serve as end products of microbial carbohydrate fermentation and as cross-feeding substrates from which other intestinal bacteria produce SCFAs such as propionate [91,92]. Pure-culture and co-culture studies further indicate that 1,2-propanediol can be produced during bacterial metabolism of fucose released from fucosylated HMOs and subsequently utilized by selected anaerobes, including *Anaerobutyricum hallii*, in pathways yielding propionate, whereas lactate and acetate can support butyrate production [93,94]. Collectively, these experimental findings indicate that HMO degradation and fermentation can alter substrate and metabolite availability within microbial communities, although the extent of these pathways in vivo is likely to depend on the strains and community members present.

Evidence for the effects of these metabolites on host intestinal responses is derived mainly from mechanistic and preclinical studies. Acetate has been implicated in epithelial energy metabolism, tight junction maintenance, and mucosal immune regulation; however, the available evidence does not establish that HMO-derived acetate improves intestinal barrier function in human infants [95]. Using immature human intestinal epithelial cells and a neonatal mouse model, Gao et al. showed that butyrate modulated barrier-related and inflammatory gene responses, including tight-junction- and mucin-related genes, under interleukin-1β stimulation [96]. These findings provide preclinical evidence for a potential barrier-regulatory effect of butyrate; however, the butyrate examined in that study was not generated through HMO fermentation, and the findings remain limited to cellular and animal models.

Human observational evidence provides complementary information on fecal metabolite dynamics during infancy. In early life, fecal fermentation profiles change markedly with dietary progression and microbiota maturation [97]. A dense longitudinal study of infant fecal metabolite dynamics identified three successive phases of fecal metabolite development: an early phase characterized by relatively low acetate and elevated succinate, an intermediate phase enriched in lactate and formate, and a later phase marked by increased propionate and butyrate concentrations [35]. Notably, the emergence of propionate- and butyrate-enriched profiles coincided with the expansion of Clostridiales-associated taxa and the gradual cessation of breastfeeding, indicating an association between dietary transition, microbial succession, and the maturation of fecal metabolic profiles rather than establishing a direct effect of HMOs [35]. A separate study of 69 mother–infant pairs combined longitudinal observational analyses of breastfeeding, fecal metabolites, and bacterial occurrence with complementary bacterial culture experiments [69]. Breastfeeding status and fecal lactate concentrations were associated with the occurrence of Peptostreptococcaceae in infant feces, while the culture experiments demonstrated taxon-specific growth responses to lactic acid, including inhibition of *Clostridioides difficile* [69]. These findings support an association between feeding status, fecal lactate, and bacterial occurrence but do not establish that HMO-derived lactate alone caused the observed microbial differences in vivo.

Aromatic lactic acids represent another class of bifidobacteria-associated metabolites for which evidence spans human observational and experimental models. In a study combining fecal microbiota and metabolite profiling in 59 healthy Danish infants with bacterial culture, monocolonized mice, and ex vivo human immune-cell experiments, breastfeeding-associated *Bifidobacterium* species converted tryptophan, phenylalanine, and tyrosine into indole-3-lactic acid, phenyllactic acid, and 4-hydroxyphenyllactic acid, respectively, through aromatic lactate dehydrogenase [98]. In infants, fecal aromatic lactic acid concentrations were associated with breastfeeding-associated bifidobacteria; experimentally, indole-3-lactic acid activated the aryl hydrocarbon receptor and hydroxycarboxylic acid receptor 3 and modulated immune-cell responses [98]. A separate study associated *Bifidobacterium infantis*-dominated infant fecal microbiota with higher indole-3-lactic acid concentrations and showed in bacterial culture and intestinal epithelial cell models that HMO-supported *B. infantis* produced indole-3-lactic acid and that this metabolite attenuated selected inflammatory responses in vitro [99]. In addition, a human supplementation study involving breastfed infants receiving *B. infantis* EVC001 reported differences in intestinal cytokine profiles, together with in vitro evidence that associated metabolites influenced T-cell polarization [100]. These studies provide human observational and intervention evidence for microbiota- and immune-related surrogate outcomes, supported by mechanistic experiments; they do not by themselves establish prevention of clinical disease in infants. Overall, the strongest evidence in this subsection concerns microbial metabolite production and fecal or immune-related surrogate outcomes, whereas direct causal evidence linking specific HMO-derived metabolites to clinical benefits in infants remains limited.

#### 4.1.3. HMO-Mediated Cross-Feeding and the Formation of Early Microbial Ecological Networks

The regulatory effects of HMOs on the infant gut microecology extend beyond the direct enrichment of dominant HMO-utilizing taxa [54]. Direct evidence for HMO-mediated cross-feeding is derived predominantly from in vitro monoculture, co-culture, and synthetic-community experiments; accordingly, the findings in this subsection primarily establish microbial mechanisms rather than in vivo ecological interactions or clinical effects. HMOs vary in chain length, glycosidic linkage, fucosylation, and sialylation, resulting in structurally diverse substrates for microbial metabolism [101]. Their utilization and the resulting ecological interactions are both HMO- and strain-dependent [72]. In a 13-member synthetic infant gut microbial community, Ioannou et al. showed that HMO-degrading strains released simpler carbohydrates, organic acids, and gases that could be utilized by other community members, while different combinations of HMOs generated distinct compositional and functional community profiles [102]. A separate seven-member synthetic-community study found that the *B. bifidum* strain included in the model was important for the formation of HMO degradation products and the support of cross-feeding. In the same model, removal of *B. longum* subsp. *infantis* was associated with an earlier onset of exponential growth and accelerated degradation of all three tested HMOs, accompanied by earlier expansion of *B. bifidum*; the authors interpreted these findings as evidence of competitive niche overlap under the experimental conditions [72]. These findings indicate that microbial roles during HMO utilization are context-dependent rather than fixed at the species level.

Within the genus *Bifidobacterium*, selected *B. bifidum* strains commonly employ extracellular glycosidases to release monosaccharides or oligosaccharide fragments that can become available to other bacteria. In a mucin-based co-culture model, Egan et al. showed that *B. breve* UCC2003 grew in the presence of the mucin-degrading strain *B. bifidum* PRL2010 by utilizing sugars released during extracellular mucin degradation [103]. Because this experiment used mucin rather than HMOs as the principal glycan source, it supports the general mechanism of glycan-derived cross-feeding but should not be interpreted as direct evidence of HMO-mediated cross-feeding. More directly, Nishiyama et al. demonstrated in vitro that extracellular sialidases from *B. bifidum* released sialic acid from sialylated oligosaccharides, including 6′-sialyllactose, and that the liberated sialic acid supported the growth of *B. breve* [104]. Together, these studies demonstrate extracellular glycan cleavage and subsequent substrate sharing among selected bifidobacterial strains under controlled experimental conditions.

HMO-mediated cross-feeding between bifidobacteria and non-bifidobacterial taxa has also been demonstrated in vitro. In single- and co-culture experiments, *B. longum* subsp. *infantis* metabolized fucosyllactose and released metabolites including lactate, acetate, and 1,2-propanediol; *A*. *hallii* subsequently utilized these intermediates and produced butyrate, propionate, and formate [94]. Although the same study assessed the occurrence of *A. hallii* using human fecal 16S rRNA gene datasets, the metabolic transfer itself was demonstrated only in culture and therefore cannot be considered direct evidence of HMO-mediated cross-feeding in vivo. Overall, the available evidence is derived predominantly from defined microbial systems involving selected strains and HMO substrates. Direct evidence confirming these metabolic interactions in vivo and their contribution to clinically relevant infant outcomes remains limited. Future studies integrating strain-resolved longitudinal sampling, stable-isotope tracing, and multi-omics approaches will help determine the prevalence and biological relevance of HMO-mediated cross-feeding in infancy.

**Figure 2:**
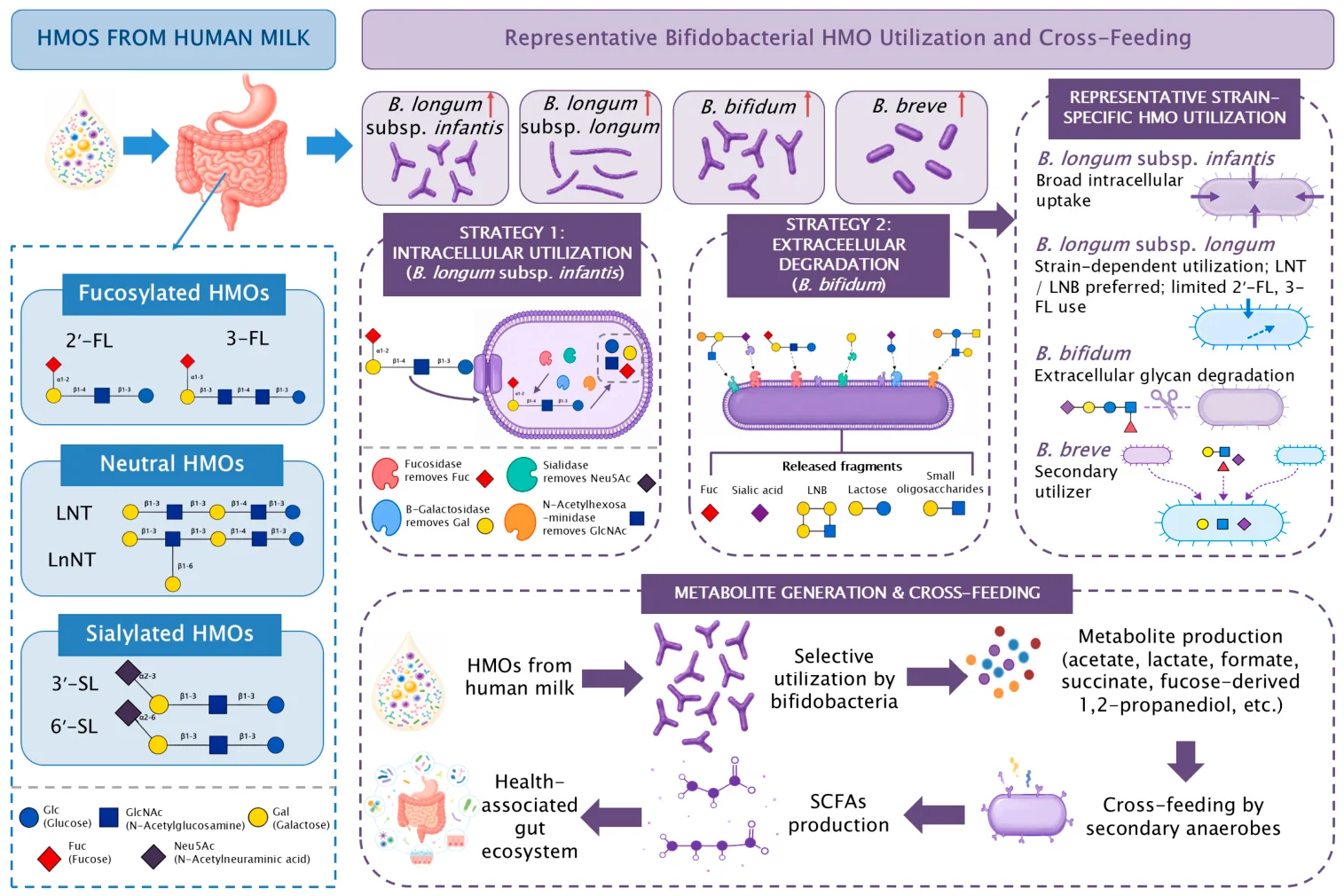
Bifidobacterial utilization of human milk oligosaccharides and HMO-driven cross-feeding networks in the infant gut. The utilization patterns shown represent generalized strategies summarized from current evidence and may vary among strains within each species. Directional arrows indicate HMO utilization, metabolite production and transfer, and microbial cross-feeding, whereas upward arrows above the bifidobacterial taxa denote generalized HMO-associated growth or enrichment that may result from direct HMO utilization or cross-feeding.

The major HMO utilization strategies and cross-feeding relationships discussed above are illustrated schematically in Figure 2, while representative experimental studies examining HMO utilization, metabolite production, and microbial cross-feeding are summarized in Table 2.

### 4.2. HMOs Shape the Gut Microecology Through Anti-Pathogen and Colonization-Resistance Mechanisms

HMOs may shape early-life microbial ecology through two conceptually distinct routes: direct interference with pathogen attachment, infectivity, growth, or biofilm formation, and indirect support of microbiota-mediated colonization resistance. Current evidence is derived mainly from in vitro, ex vivo, and preclinical animal studies. These studies establish biological plausibility and, in some cases, causal relationships within experimental systems but should not be interpreted as direct evidence that HMOs prevent clinically diagnosed infections in human infants. Direct human intervention evidence remains limited.

Rotavirus and norovirus are major causes of acute gastroenteritis, particularly in infants and young children [105]. Evidence for direct antiviral effects of HMOs is derived mainly from in vitro infectivity and structural binding studies. In MA104 cell assays, Laucirica et al. tested 2′-FL, 3′-SL, 6′-SL, and galacto-oligosaccharides against the human rotavirus strains G1P[8] and G2P[4] [106]. All four oligosaccharides reduced viral infectivity under at least some experimental conditions, although the magnitude of inhibition depended on the viral genotype, oligosaccharide structure, concentration, and timing of exposure. The maximum reduction in G1P[8] infectivity was observed with 2′-FL added after the onset of infection, whereas the combination of 3′-SL and 6′-SL produced the greatest reduction in G2P[4] infectivity. Pretreatment experiments indicated that the reduction in infectivity was mediated primarily through effects on viral particles rather than through pretreatment of host cells [106]. These findings demonstrate that HMOs exhibit structure- and strain-dependent antiviral activity in cell-culture systems.

Using norovirus capsid proteins, virus-like particles, surrogate histo-blood group antigen (HBGA) ligands, and X-ray crystallography, Weichert et al. showed that the fucosylated HMOs 2′-FL and 3-FL occupied HBGA-binding pockets on the GII.10 norovirus capsid and inhibited capsid binding to surrogate HBGA samples [107]. These structural and biochemical findings support a molecular-decoy mechanism in which selected HMOs may compete with host glycans for viral binding sites. Nevertheless, HBGA-binding inhibition is a surrogate endpoint obtained without assessment of live-virus infection or clinically diagnosed norovirus gastroenteritis.

Preclinical evidence also suggests that dietary HMOs can modify immune responses during rotavirus exposure. In a neonatal piglet study, colostrum-deprived animals received unsupplemented formula, formula containing four HMOs plus free sialic acid, or formula with non-HMO prebiotics; half were challenged with porcine rotavirus [108]. Regardless of infection status, piglets receiving HMOs plus free sialic acid had higher circulating natural killer-cell and mesenteric-lymph-node effector-memory T-cell populations than unsupplemented controls, while noninfected piglets also showed more IFN-γ-producing peripheral blood mononuclear cells [108].

Evidence for direct antibacterial and antibiofilm effects is likewise derived mainly from in vitro studies. Ackerman et al. examined HMOs isolated from individual donor milk samples and found that selected HMO mixtures modified the growth, biofilm formation, and cellular organization of *Streptococcus agalactiae* (group B streptococcus [GBS]) under controlled culture conditions. The magnitude and direction of these effects varied according to the donor HMO profile and bacterial strain [109]. In a subsequent study involving HMO preparations from 14 additional donors, three GBS strains, methicillin-resistant *Staphylococcus aureus* USA300, and *Acinetobacter baumannii*, selected HMO preparations reduced GBS growth and biofilm formation and reduced *S. aureus* biofilm formation under specific culture conditions. Maximum reductions of approximately 93% in GBS biofilm formation and 60% in *S. aureus* biofilm formation were observed for individual donor preparations; however, the effects were strongly donor-, strain-, and medium-dependent, and some HMO preparations increased rather than decreased biofilm production [110]. These findings therefore support context-dependent antimicrobial and antibiofilm activity rather than a uniform inhibitory effect across HMOs or bacterial strains.

Jarzynka et al. extended this evidence by testing pooled and fractionated HMOs against planktonic cultures and mature 48 h biofilms formed by four Gram-negative and three Gram-positive pathogen species. The most consistent activity was observed against Gram-positive organisms, particularly *Enterococcus faecalis*, whereas little or no comparable activity was detected against the tested Gram-negative species under planktonic conditions. Treatment of mature biofilms reduced viable-cell counts in selected *S. aureus*, *E. faecalis*, and *Enterococcus faecium* isolates, but the responses varied by species, isolate, HMO fraction, and concentration [111]. Because these experiments measured bacterial growth and biofilm viability in vitro, they do not by themselves demonstrate reduced intestinal colonization, persistence, or infection in infants.

Evidence beyond bacterial culture is available from reproductive-tract models of GBS infection. Moore et al. combined a pregnant-mouse model of ascending infection with human EpiVaginal tissue and ex vivo human gestational-membrane models. HMO supplementation reduced GBS adherence to human tissue models and decreased bacterial burdens, inflammatory responses, and selected adverse pregnancy outcomes in infected mice [112]. These findings provide preclinical and ex vivo evidence that HMO mixtures can influence bacterial adherence and tissue infection.

Indirect microbiota-mediated colonization resistance remains biologically plausible because HMOs selectively support HMO-utilizing bifidobacteria and contribute to the production of organic acids and other metabolites that can alter nutrient availability, environmental pH, and interspecies competition, as discussed in Section 4.1.1, Section 4.1.2 and Section 4.1.3. However, increased abundance in fecal samples should not be equated automatically with stable intestinal colonization, and the resulting ecological changes have not yet been shown conclusively to cause lower rates of clinically confirmed infection in infants. Overall, current evidence supports structure-, pathogen-, strain-, concentration-, and model-dependent antiviral, antiadhesive, antimicrobial, antibiofilm, and immunomodulatory activities of HMOs. Well-controlled human intervention studies with clearly defined HMO exposures and clinically verified infection outcomes are required to determine whether these experimental mechanisms translate into meaningful protection in infants.

Representative preclinical and human studies on barrier, immune, and anti-pathogen effects relevant to HMOs and microbial metabolites are summarized in Table 3.

## 5. Conclusions

Early-life nutrition is closely linked to the establishment and maturation of the infant gut microbiota, a process that has important implications for metabolic, epithelial, and immune development. Human milk, particularly its bioactive components such as HMOs, plays a central role in shaping a milk-adapted microbial ecosystem by selectively supporting beneficial taxa, promoting microbial metabolic activity, and contributing to host–microbe homeostasis. A clearer understanding of these nutrition–microbiota interactions may help define critical windows for early dietary modulation, refine breastfeeding and mixed-feeding guidance, improve the use of donor human milk, and support the rational design of infant formulas supplemented with HMOs, probiotics, prebiotics, or synbiotics. From a public health perspective, such knowledge may inform early-life strategies intended to support microbial, immune, and metabolic development and may help clarify whether microbiota-directed nutritional approaches can contribute to lower risks of infection and later-life non-communicable diseases. Looking forward, microbiome-based early-life interventions should move from broad taxonomic associations toward mechanism-driven, equitable, and individualized strategies. This will require longitudinal birth cohorts, accurate assessment of feeding exposure, coordinated sampling of maternal, infant, and household environmental microbial reservoirs, strain-resolved metagenomics, glycomics, metabolomics, and well-controlled intervention studies to clarify causal pathways, evaluate safety and efficacy, and develop scalable nutritional approaches that support infant health and long-term disease prevention.

## Figures and Tables

**Table 1:** Summary of human studies on early-life gut microbiota succession and infant feeding, human milk, or HMO exposures.

Study	Population, Design, and Sample Size	Exposure, Dose, and Comparator	Age and Follow-Up	Microbiome, Metabolome, or Exposure-Assessment Method	Outcome Type and Main Findings
Bäckhed et al. (2015) [4]	Swedish mother-infant cohort; prospective longitudinal study; *n* = 98 mother-infant pairs.	Vaginal versus cesarean delivery; breastfeeding status and cessation.	Maternal stool at delivery; infant stool at approximately 4 days, 4 months, and 12 months.	Shotgun metagenomic sequencing with strain-level mother-infant comparisons.	Microbiome surrogate: Vaginally delivered neonates showed substantially greater early resemblance to the maternal gut microbiota than cesarean-born neonates. Maternal strains were preferentially retained, and cessation of breastfeeding was a major transition toward an adult-like microbiota.
Roswall et al. (2021) [5]	Swedish children; prospective longitudinal cohort; *n* = 471.	Age, feeding, and early-life lifestyle/environmental factors; no fixed intervention dose.	Samples at 4 and 12 months and at 3 and 5 years.	16S rRNA gene profiling with longitudinal trajectory analysis.	Microbiome surrogate: Bacterial genera followed distinct colonization trajectories, including early, transitional, and late colonizers. Later colonizers such as *Methanobrevibacter* and Christensenellaceae were associated with increasing alpha-diversity, while trajectories remained strongly individualized.
Selma-Royo et al. (2024) [9]	Spanish MAMI mother-infant cohort; prospective longitudinal study; *n* = 34 mother-infant pairs (home birth *n* = 10, hospital vaginal birth *n* = 13, hospital cesarean birth *n* = 11).	Place and mode of birth; breastfeeding duration and practices; no fecal microbiota transplantation intervention.	Infant samples at 1 week and 1, 6, and 12 months.	Shotgun metagenomics with strain-resolved mother-infant transmission analysis.	Microbiome surrogate: Place and mode of birth influenced the timing and persistence of maternally transmitted strains. Breastfeeding duration modified retention and functional diversity of selected transmitted strains, including *Bifidobacterium longum*.
Sindi et al. (2024) [10]	Australian lactating mothers; single-arm pilot dietary intervention; *n* = 11.	Two-week diet lower in fat and sugar and higher in fiber; no parallel control group.	Pre-intervention, immediately post-intervention, and follow-up through 8 weeks after the intervention.	Full-length 16S rRNA gene sequencing of human milk and maternal fecal samples.	Microbiome surrogate: The short dietary intervention was associated with changes in human milk microbiota, including increased *Cutibacterium acnes* and decreased *Haemophilus parainfluenzae*. Some changes persisted during follow-up; the small uncontrolled pilot design limits causal inference.
Butts et al. (2020) [11]	Breastfeeding women and their infants in New Zealand; cross-sectional study; *n* = 78 women-infant pairs.	Maternal ethnicity, body mass index, and delivery mode; observational exposures.	Approximately 6–8 weeks postpartum.	16S rRNA gene sequencing of human milk and fecal samples; human milk immune-protein assays.	Microbiome and immune surrogates: Human milk microbiota and selected immune proteins differed across ethnic groups. Maternal body mass index was not consistently associated with milk or infant fecal microbiota; observed associations do not establish causal effects of ethnicity or adiposity.
Ferretti et al. (2018) [19]	Italian mother-infant pairs; prospective longitudinal multi-body-site study; *n* = 25 pairs.	Maternal gut, skin, oral, vaginal, and milk microbial sources; observational vertical transmission.	Birth through 4 months.	Strain-resolved shotgun metagenomics of maternal body sites and infant feces.	Microbiome surrogate: Maternal gut strains contributed the largest and most persistent transmitted fraction, whereas skin and vaginal strains were generally more transient. Microbial acquisition from maternal sources continued after birth.
Dubois et al. (2024) [20]	Finnish/European longitudinal cohorts: HELMi *n* = 74 infants and SECFLOR *n* = 7 cesarean-born infants receiving maternal fecal microbiota transplantation.	Paternal and household microbial contact; cesarean birth; maternal fecal microbiota transplantation in SECFLOR.	Birth through the first year of life.	Strain-resolved shotgun metagenomics of infant and family-member samples.	Microbiome surrogate: Fathers were a stable source of infant gut strains, with cumulative paternal contributions approaching maternal contributions by 1 year. Maternal fecal microbiota transplantation increased mother-infant strain sharing in cesarean-born infants.
Leech et al. (2024) [21]	Australian mother-infant dyads; prospective sub-study; *n* = 25 (vaginal/no intrapartum antibiotics *n* = 9, vaginal/antibiotics *n* = 7, cesarean/antibiotics *n* = 9).	Delivery mode and intrapartum antibiotic exposure.	Maternal and infant sampling at approximately 6 weeks postpartum.	Shotgun metagenomic sequencing of maternal and infant stool.	Microbiome surrogate: At 6 weeks, delivery mode was a stronger determinant of infant gut microbiome composition than intrapartum antibiotic exposure. Cesarean-born infants showed decreased representation of Bacteroidota.
Shao et al. (2019) [22]	UK prospective cohort of full-term infants; *n* = 596 infants with 1679 stool samples; paired maternal analyses were performed in a reported subset.	Cesarean versus vaginal delivery; intrapartum antibiotics; neonatal feeding.	Neonatal period with longitudinal follow-up during infancy.	Whole-genome shotgun metagenomics, bacterial culture, and isolate/strain sequencing.	Microbiome surrogate: Cesarean delivery was associated with reduced transmission of maternal commensals, particularly *Bacteroides*, and increased colonization by hospital-associated opportunistic pathogens including *Enterococcus*, *Enterobacter*, and *Klebsiella*.
Shao et al. (2024) [23]	UK longitudinal neonatal metagenomic cohort; *n* = 1288 infants.	Delivery mode and maternal/infant host factors, including feeding-related exposures; observational comparisons.	Neonatal period with longitudinal infant sampling.	Longitudinal fecal metagenomics with experimental validation in germ-free mice.	Microbiome surrogate with preclinical validation: Three neonatal community states were identified. An *Enterococcus faecalis*-dominated state carried persistent pathogen burdens, whereas *Bifidobacterium longum*- and *B. breve*-dominated states showed greater colonization resistance in experimental validation.
Azad et al. (2016) [24]	Canadian CHILD cohort; prospective study; *n* = 198 healthy term infants.	Intrapartum antibiotic exposure, delivery mode, and breastfeeding.	Infant stool at 3 and 12 months.	16S rRNA gene sequencing of fecal samples.	Microbiome surrogate: Intrapartum antibiotics were associated with altered infant gut microbiota, with effects modified by delivery mode and breastfeeding. Some emergency-cesarean-associated differences persisted to 12 months among non-breastfed infants.
Timmerman et al. (2017) [27]	Dutch infants; densely sampled observational longitudinal study; *n* = 8 (breastfed *n* = 4, formula-fed *n* = 4).	Breastfeeding versus formula feeding; no assigned intervention.	First 12 weeks of life; 17 consecutive sampling points.	16S rRNA gene sequencing and quantitative PCR.	Microbiome surrogate: Early communities progressed from birth/skin-associated taxa toward communities frequently dominated by Bifidobacteriaceae. Interindividual variation was substantial and was particularly pronounced among breastfed infants.
Beller et al. (2021) [28]	Densely sampled Belgian infant cohort; *n* = 8 infants with 303 longitudinal samples; comparison with an external adult Flemish Gut Flora Project (FGFP) reference cohort, *n* = 1106.	Developmental stage and early-life exposures; observational analysis.	First year of life.	Metagenomic analysis of bacterial and viral community development.	Microbiome surrogate: Infant gut ecosystems progressed through reproducible successional stages, while the timing and composition of transitions remained individual-specific. The study primarily supports ecological trajectory patterns rather than a specific feeding effect.
Olm et al. (2022) [29]	Hadza deep-metagenomic analysis: 39 infant fecal samples, with corresponding maternal samples for 23 infants; global meta-analysis: 1900 infant fecal 16S rRNA profiles from 18 populations.	Hunter-gatherer versus industrialized lifestyle and population-associated feeding/environmental context; observational comparison.	Infancy; age windows varied across the global datasets.	Deep shotgun metagenomics, genome reconstruction, and global comparative 16S rRNA analysis.	Microbiome surrogate: Infant microbiome assembly differed across lifestyles. Hadza infants carried extensive previously uncharacterized microbial diversity, whereas industrialized infants had lower carriage of *Bifidobacterium infantis* and human-milk-utilization genes.
Fahur Bottino et al. (2025) [30]	Global longitudinal data from 12 countries; *n* = 1827 infants and 3154 samples.	Geographic, feeding, environmental, and lifestyle variation; observational comparisons.	Birth to approximately 18 months, analyzed in age windows of about 3 months.	Metagenomic taxonomic profiling and machine-learning trajectory analysis.	Microbiome surrogate: Common age-related succession patterns were observed across populations, but feeding, environmental, and lifestyle context substantially modified community assembly and the timing of microbial transitions.
Tsukuda et al. (2021) [35]	Japanese infants; dense longitudinal cohort; *n* = 12 infants and 1048 fecal samples.	Breastfeeding, weaning, and dietary transition; observational longitudinal exposures.	Birth through 2 years.	Fecal microbiota analysis and targeted/untargeted metabolite profiling, including short-chain fatty acids.	Metabolic and microbiome surrogates: Fecal metabolites followed successive phases, from early succinate enrichment to lactate/formate and later propionate/butyrate enrichment. Later-phase metabolites coincided with Clostridiales expansion and reduced breastfeeding, supporting association rather than a single causal dietary effect.
Vatanen et al. (2018) [44]	Multinational TEDDY cohort; prospective longitudinal study; *n* = 783 children and 10,913 fecal metagenomes.	Breastfeeding, antibiotics, probiotics, geographic context, and progression to islet autoimmunity/type 1 diabetes; observational exposures.	Monthly sampling from 3 months through study follow-up in early childhood.	Shotgun metagenomics with taxonomic and functional profiling.	Microbiome surrogate: The infant microbiome was highly dynamic and individualized. First-year communities were often dominated by one of several *Bifidobacterium* species or Proteobacteria, and *B. longum* HMO-utilization genes were enriched in breastfed infants.
Rumbold et al. (2025) [49]	Australian moderate-to-late preterm infants; multicenter blinded randomized clinical trial; *n* = 201 (donor milk *n* = 99, formula *n* = 102).	Supplemental pasteurized donor human milk versus term infant formula when mother’s own milk was insufficient; supplementation for up to 8 days.	Enrollment within 4 days after birth; clinical follow-up through 6 months corrected age.	Clinical feeding and safety outcomes, including time to full enteral feeding and later growth/health assessments.	Clinical outcome: Donor milk and formula were compared as short-term supplements. The primary outcome was time to full enteral feeding; this trial did not provide a microbiome endpoint and should not be interpreted as direct evidence of microbiota modulation.
Pärnänen et al. (2022) [55]	Finnish neonatal metagenomic dataset; *n* = 46 infants, with validation using additional public datasets.	Formula feeding versus human-milk exposure; preterm/full-term status.	Neonatal period; exact sampling ages varied by cohort.	Shotgun metagenomics for microbial taxa and antibiotic-resistance genes.	Microbiome surrogate: Formula feeding was associated with altered community composition and a higher antibiotic-resistance-gene burden, particularly among facultative anaerobes. Observational design and cohort heterogeneity limit causal interpretation.
Wang et al. (2025) [57]	Chinese infants in a randomized feeding study; synbiotic formula *n* = 112, prebiotic formula *n* = 112, breastfed reference *n* = 60.	Synbiotic formula containing scGOS/lcFOS plus *B. breve* M-16V versus prebiotic-only formula; breastfed nonrandomized reference.	Baseline, 17 weeks, and 12 months.	16S rRNA gene sequencing; fecal pH, short-chain fatty acids, lactic acid, and secretory IgA.	Microbiome and immune surrogates: Among cesarean-born infants, synbiotic formula, but not prebiotic-only formula, increased bifidobacterial diversity and shifted selected taxa toward profiles observed in vaginally born infants. Some microbiome differences persisted after the intervention.
Estorninos et al. (2022) [58]	Healthy term Filipino infants; double-blind randomized formula trial with a nonrandomized human-milk-fed reference. The parent formula trial randomized *n* = 230 (control formula *n* = 115; test formula *n* = 115). The microbiome/immune analysis reported control formula *n* = 112, test formula *n* = 114, and human-milk-fed reference *n* = 70.	Standard formula versus formula containing 7.2 g/L bovine milk-derived oligosaccharides; human-milk-fed reference group.	Enrollment at 21–26 days; assigned formula through 6 months; fecal samples at baseline and 2.5 and 4 months.	16S rRNA-based microbiota analysis, fecal metabolites, and intestinal immune biomarkers.	Microbiome and immune surrogates: Oligosaccharide-supplemented formula shifted microbiota composition toward the human-milk-fed reference, increased bifidobacterial abundance, and altered selected intestinal immune-defense markers. These were surrogate rather than clinical endpoints.
Zuffa et al. (2025) [59]	Randomized double-blind feeding study; parent cohort *n* = 311 (control formula *n* = 108, test formula *n* = 115, breastfed reference *n* = 88); microbiome/metabolome analysis subset *n* = 164.	Standard formula versus formula containing large milk phospholipid-coated lipid droplets; breastfed nonrandomized reference.	Intervention through 17 weeks; assessments through 12 months.	Fecal microbiome and metabolome profiling, plasma metabolomics, and body-composition assessment.	Microbiome and metabolic surrogates: The test formula was associated with lower abundance of selected opportunistic taxa and enrichment of selected butyrate-associated taxa, with multi-omic trajectories closer to the breastfed reference. Findings from the analyzed subset should not be generalized to all randomized participants without qualification.
Wang et al. (2025) [60]	STRONG Kids 2 cohort; cross-sectional analysis at 6 weeks; *n* = 75 infants (exclusive breastfeeding *n* = 25, exclusive formula feeding *n* = 25, mixed feeding *n* = 25).	Exclusive breastfeeding, exclusive formula feeding, or mixed feeding; delivery mode considered as a covariate.	6 weeks of age.	Shotgun metagenomics and untargeted fecal metabolomics.	Microbiome and metabolic surrogates: Mixed-fed infants differed from exclusively breastfed infants in community diversity, functional pathways, and metabolite profiles, but were more similar to formula-fed infants. The cross-sectional design supports association, not proof that formula exposure caused all observed differences.
Myers et al. (2026) [61]	Two prospective European birth cohorts; combined *n* = 519 infants.	Breastfeeding with versus without formula supplementation; amount, timing, and composition of supplemental formula.	Assessment at approximately 3 months.	Plasma lipidomics and stool shotgun metagenomics.	Microbiome and metabolic surrogates: Formula supplementation during breastfeeding was associated with detectable shifts in plasma lipids and gut microbiota. Mixed feeding did not form a uniform intermediate phenotype; associations varied with formula amount, timing, and composition.
Zhang et al. (2026) [68]	Multi-ethnic Asian GUSTO mother-offspring cohort; *n* = 248 mothers with HMO quantification (*n* = 205 at 3 weeks; *n* = 114 at 3 months).	Maternal ethnicity, *FUT2* genotype/secretor status, gestational age, and lactation stage; no assigned HMO dose.	Human milk collected at 3 weeks and 3 months postpartum.	High-performance liquid chromatography quantification of 19 major HMOs.	Exposure-assessment outcome: HMO composition was broadly similar across Chinese, Malay, and Indian participants, while *FUT2* variation strongly determined secretor-associated profiles. Preterm delivery and lactation stage were associated with selected HMO differences; this study measured exposure composition rather than infant outcomes.
Huertas-Díaz et al. (2023) [69]	Swiss CARE birth cohort; longitudinal observational study; *n* = 69 mother-infant pairs, with complementary in vitro bacterial assays.	Breastfeeding status, fecal lactate, and human-milk HMO composition; no assigned lactate or HMO dose in the human cohort.	Three early-life sampling points; feeding classification defined at 4 months.	Quantitative microbiota profiling, HPLC with refractive-index detection, proton NMR metabolomics, and bacterial culture assays.	Microbiome and metabolic surrogates: Breastfeeding status and fecal lactate were associated with the occurrence of Peptostreptococcaceae. In vitro assays showed taxon-specific responses to lactate, but the study did not establish that HMO-derived lactate alone caused the in vivo microbial differences.

**Table 2:** Summary of experimental studies on HMO utilization, metabolite production, and microbial cross-feeding.

Study	Experimental Model, Design, and Sample Size/Replicates	HMO or Carbohydrate Substrate and Dose	Experimental Method	Mechanistic Outcome(s)	Main Finding
De Bruyn et al. (2024) [71]	Ex vivo colonic incubation models seeded with fecal microbiota from the same six breastfed children sampled at approximately 3 months and again at approximately 12 months; paired-donor design, *n* = 6 at each age stage.	Age-adapted six-HMO blend at 2.5 g/L, with or without *B. longum* subsp. *infantis* LMG 11588; matched controls without the combined intervention.	Anaerobic ex vivo colonic incubation with HMO-consumption and short-chain-fatty-acid analyses.	HMO disappearance and production of acetate, lactate, propionate, and butyrate.	The HMO blend was consumed more rapidly and completely when combined with *B. infantis*. Metabolite production from the combination exceeded the arithmetic sum of the individual interventions, supporting ex vivo metabolic synergy; clinical benefit was not assessed.
Pröschle-Donoso et al. (2025) [72]	Seven-member synthetic infant gut microbial community; single-species dropout design; eight anaerobic batch-bioreactor experiments conducted in duplicate.	Total HMO concentration 2%: 45% lacto-*N*-tetraose, 40% 2′-fucosyllactose, and 15% 3′-sialyllactose.	Anaerobic batch bioreactors, quantitative PCR, HMO and organic-acid measurements, metatranscriptomics, and ordinary-differential-equation modeling.	Community growth, species abundance, HMO degradation, metabolite production, transcriptional responses, and interspecies interactions.	*B. bifidum* contributed substantially to extracellular HMO degradation and resource provision. Removal of *B. longum* subsp. *infantis* accelerated community growth and degradation of all three HMOs, indicating competitive niche overlap under the experimental conditions.
Sela et al. (2008) [75]	*B. longum* subsp. *infantis* ATCC 15697 in a genome-centered mechanistic study with HMO-growth, proteomic, metabolomic, and stable-isotope-tracing experiments. HMO growth assays were performed in three biologically independent replicates. The proteomic dataset was reported for two samples; the independent biological-replicate number for the isotope-tracing experiment was NR.	Pooled HMOs at 2% (*w*/*v*) in semisynthetic MRS medium for growth assays and modified MRS medium for proteomic cultures; cultures were grown anaerobically at 37 °C. Stable-isotope experiments used a defined medium containing 0.2% ^13^C-labeled carbon source; chase experiments added 100 µL of a 0.2% unlabeled chase-sugar solution to each 6-mL aliquot.	Complete-genome sequencing and comparative genomics, HMO growth assays, proteomic and metabolite profiling, and stable-isotope tracing.	Genomic capacity for HMO uptake and catabolism and associated fermentative responses.	An approximately 43-kbp gene cluster encoded transporters, solute-binding proteins, and glycosidases consistent with extensive HMO utilization. The study established genomic and biochemical capacity in a model strain, not in vivo infant colonization or clinical benefit.
LoCascio et al. (2010) [76]	Fifteen *B. longum* strains representing different subspecies, host origins, and HMO-growth phenotypes; comparative genomic hybridizations for each strain were performed in two biological replicates.	Human-milk oligosaccharides at 1.5% (*w*/*v*) as the sole carbon source.	Comparative genomic hybridization, multilocus sequence typing, and in vitro growth-phenotype comparison.	Conservation of HMO-utilization gene regions and strain/subspecies-specific growth phenotypes.	HMO-utilization gene regions were conserved in HMO-growing *B. longum* subsp. *infantis* strains but were absent or divergent in many *B. longum* subsp. *longum* strains. The findings support strain-level heterogeneity rather than a species-wide HMO phenotype.
Gotoh et al. (2018) [79]	Four *B. bifidum* strains were evaluated in strain-level HMO-degradation and cross-feeding assays, and fecal cultures included five donors (one infant, two children, and two adults). Strain cultures and fecal-culture experiments were performed in three independent replicates.	Basal medium containing 1% (*w*/*v*) total HMOs was used for the strain-level and fecal-culture experiments; 1% (*w*/*v*) LNT was used in the complementary intra-species cross-feeding assay.	In vitro fecal culture, strain supplementation, and oligosaccharide/degradation-product analysis.	Release and sharing of HMO-derived sugars and growth of other bifidobacterial taxa.	*B. bifidum* strains released extracellular HMO-degradation products that became available to other bifidobacteria and supported community growth. The extent of sharing was strain- and community-dependent.
Ward et al. (2007) [80]	Five bifidobacterial strains: *B. longum* subsp. *infantis*, *B. bifidum*, *B. longum* subsp. *longum*, *B. breve*, and *B. adolescentis*; cultures were performed in triplicate.	Pooled human-milk oligosaccharides at 1% (*w*/*v*) as the sole fermentable carbohydrate.	In vitro growth, thin-layer chromatography of spent media, and monosaccharide-fermentation tests.	Cell growth and patterns of intact-HMO degradation or released monosaccharides.	*B. longum* subsp. *infantis* showed the strongest growth on pooled HMOs. *B. bifidum* released free sialic acid, fucose, and *N*-acetylglucosamine, whereas the other strains showed more limited utilization, demonstrating species- and strain-dependent strategies.
Thongaram et al. (2017) [82]	Twenty-four strains (12 lactobacilli and 12 bifidobacteria), including commercial probiotic strains; at least three independent replicates were completed for each carbohydrate, and postfermentation HPLC/TLC analyses used three independent replicates.	3′-sialyllactose, 6′-sialyllactose, 2′-fucosyllactose, 3-fucosyllactose, lacto-*N*-neotetraose, and constituent monosaccharides, each supplied at 1% (*w*/*v*); cultures were inoculated at 1% (*v*/*v*).	High-throughput low-volume growth assays and HPLC-based substrate-consumption analysis.	Strain-specific growth and consumption of individual HMOs.	HMO utilization was highly strain specific. *B. longum* subsp. *infantis* ATCC 15697 and *B. infantis* M-63 used the broadest panel; *B. infantis* M-63 consumed most 2′-FL while releasing fucose, and LNnT utilization was observed in selected *B. infantis* and *B. breve* strains.
Walsh et al. (2022) [86]	Four infant-derived bifidobacterial strains in single and mixed cultures: *B. bifidum* R0071, *B. breve* M-16V, *B. infantis* R0033, and *B. infantis* M-63. Culture experiments were performed in biological triplicate; reported quantitative analyses used technical duplicates.	2′-fucosyllactose, 1.2 g/L; modeled non-secretor HMO fraction, 3.8 g/L; modeled secretor HMO fraction, 5.0 g/L.	Single- and co-culture growth, HPLC substrate analysis, strain-specific quantitative PCR, and metabolomics.	Community growth, relative abundance, HMO use, and production of acetate, lactate, formate, and 1,2-propanediol.	Mixed cultivation increased total bifidobacterial growth and enabled *B. breve* expansion despite limited HMO assimilation in monoculture, supporting metabolite/substrate sharing. Magnitudes depended on the strain combination and milk-oligosaccharide profile.
Van den Abbeele et al. (2021) [87]	Short-term incubations used fecal microbiota from five 3-month-old breastfed-infant donors and five 2–3-year-old toddler donors. One representative donor from each age group was selected for the 7-week M-SHIME experiment.	2′-fucosyllactose versus lactose at 5 g/L in the 48-h short-term incubations. During the 3-week M-SHIME treatment period, each carbohydrate was added at 10 g/L to the nutritional medium, yielding approximately 7 g/L in the suspension entering the proximal-colon compartment.	Forty-eight-hour fecal incubations and a 7-week mucosal simulator of the human intestinal microbial ecosystem (M-SHIME) with microbial and metabolite profiling.	Microbial composition, short-chain and branched-chain fatty acids, lactate, pH, and gas production.	2′-FL increased Bifidobacteriaceae and altered fermentation products in both infant and toddler microbiota, including increased acetate and selected downstream short-chain fatty acids and decreased branched-chain fatty acids. These are simulator outcomes and not direct infant clinical effects.
Li et al. (2022) [89]	Twelve full-term healthy infants were screened, and seven fecal samples with >30% relative abundance of *B. longum* were selected as independent anaerobic-fermentation inocula. The seven donor inocula constituted the biological replicate set (*n* = 7).	Six individual HMOs—2′-FL, 3-FL, 3′-SL, 6′-SL, LNT, and LNnT—were each added at 50 mg dry matter to 3 mL buffer plus 1 mL fecal slurry, corresponding to an approximate final concentration of 12.5 mg/mL or 1.25% (*w*/*v*). FOS and GOS were used as positive controls, and a no-substrate tube was used as the negative control.	Individual-inoculum in vitro fermentation with microbial-composition, organic-acid, and gas analyses.	Acetate, lactate, gas, and changes in taxon relative abundance.	All six HMOs produced broadly similar acetate concentrations, while neutral HMOs produced more lactate than sialylated HMOs. Responses varied among individual inocula; FOS promoted *Klebsiella pneumoniae* in some cultures, underscoring donor-specific effects.
Zabel et al. (2019) [90]	*B. longum* subsp. *infantis* Bi-26 was grown in batch culture with 2′-FL or lactose. For RNA-seq, three separate cultures were prepared per carbon source, with one culture harvested at each of the early-, mid-log-, and late-log phases; therefore, each carbon-source-by-growth-phase condition was represented by one biological culture. Metabolite analysis used five biological replicate cultures, and each analytical sample was analyzed in triplicate.	2′-fucosyllactose at 1% (*w*/*v*), with lactose at 1% (*w*/*v*) as the comparator carbon source.	RNA-seq transcriptomics and NMR/GC-MS metabolomics across growth phases.	Expression of transport/catabolic genes and production of formate, acetate, lactate, and 1,2-propanediol.	Growth on 2′-FL upregulated multiple carbohydrate-transport and catabolic clusters, including previously uncharacterized ABC-type systems, and generated metabolites consistent with intracellular fucose cleavage. This is strain-specific mechanistic evidence.
Dedon et al. (2020) [93]	*B. longum* subsp. *infantis* ATCC 15697 in single-substrate and co-fermentation experiments; microplate growth assays were performed in biological triplicate with three technical replicates, and large-volume metabolic profiling used 15 replicate cultures.	0.8% free L-fucose, 0.8% 2′-FL, and co-fermentation conditions containing 0.8% fucose with limiting lactose or glucose.	Growth, targeted metabolite measurements, transcriptomics, proteomics, and co-fermentation analyses.	Biomass formation, fucose-pathway expression, and secretion of 1,2-propanediol.	Free fucose and 2′-FL-derived fucose entered a common catabolic pathway leading to 1,2-propanediol. Co-fermentation with limiting lactose or glucose increased biomass, demonstrating how additional carbon sources affect fucose utilization.
Schwab et al. (2017) [94]	*B. longum* subsp. *infantis* and *A. hallii* in single- and co-culture experiments; growth experiments were performed in at least three independent replicates, except *B. longum* subsp. *infantis* growth with glucose or L-fucose, which was assessed in duplicate; age-prevalence analysis used 857 fecal 16S rRNA gene libraries.	Modified YCFA medium containing 50 mM glucose, 40 mM L-fucose, or a fucosyllactose mixture of 6 mM 2′-FL plus 6 mM 3′-FL.	Anaerobic cultivation, metabolite analysis, and 16S rRNA gene dataset screening.	Cross-feeding of 1,2-propanediol, acetate, and formate and downstream butyrate/propionate production.	*B. infantis* converted fucose/fucosyllactose into 1,2-propanediol, acetate, and formate; *A. hallii* used these intermediates to generate butyrate- and propionate-related products. The age analysis supported later emergence of *A. hallii* but did not demonstrate this interaction directly in vivo.
Ioannou et al. (2024) [102]	BIG-Syc synthetic infant gut community comprising 13 infant-derived strains. Continuous bioreactors were run in triplicate. The four-HMO condition was evaluated in two independent runs, yielding six fermentor replicates in total, whereas the five-HMO condition was evaluated in one run with three fermentor replicates.	Basal medium containing 0.4% (*w*/*v*) HMO mixture. The four-HMO mixture contained 2′-FL at 39.2 ± 5%, 3-FL at 23.5 ± 3%, 3′-SL at 9.8 ± 3%, and 6′-SL at 27.5 ± 3% of dry weight. The five-HMO mixture contained 2′-FL at 52 ± 5%, 3-FL at 13 ± 3%, 3′-SL at 4 ± 1%, 6′-SL at 5 ± 1%, and LNT at 26 ± 3% of dry weight.	Anaerobic continuous fermentation with quantitative community profiling, metabolomics, metaproteomics, and genome-scale resource-sharing analysis.	Community composition, HMO-dependent resource flow, and metabolite/protein profiles.	BIG-Syc reproduced selected features of infant gut communities, and different HMO mixtures generated distinct compositional and functional profiles. In the four-HMO condition, *B. infantis* dominance was observed in four of six replicates, whereas the five-HMO mixture favored a more diverse profile with *B. bifidum* prominence. Resource sharing and mutual suppression were specific to the synthetic-community conditions.
Egan et al. (2014) [103]	*B. breve* UCC2003 co-cultured with the mucin-degrading strain *B. bifidum* PRL2010; growth experiments were performed in duplicate.	Mucin at 0.4% (*w*/*v*); direct HMO exposure NA.	Co-culture growth, HPAEC-PAD carbohydrate analysis, and transcriptome analysis.	*B. breve* viability/growth and utilization of sugars released by extracellular mucin degradation.	*B. breve* grew in mucin medium only in the presence of *B. bifidum*, supporting cross-feeding on released glycans. Because the principal substrate was mucin rather than HMOs, this is evidence for glycan-derived cross-feeding generally, not direct HMO-mediated cross-feeding.
Nishiyama et al. (2018) [104]	*B. bifidum* ATCC 15696 wild type and the Δ*siabb2* mutant were evaluated in single- and co-culture experiments with *B. breve* JCM 7019 and, in selected assays, *B. longum* subsp. *infantis* JCM 1222. The principal single-culture 6′-SL growth experiment used *n* = 5; principal co-culture, recombinant-sialidase, and mucin-growth experiments used *n* = 3; *B. breve nan*-gene-expression experiments used *n* = 5. In the original article, *n* denotes the number of independent experiments.	Growth assays used 0.5% (*w*/*v*) 6′-SL, porcine colonic mucin, lactose, or *N*-acetylneuraminic acid; co-culture medium additionally contained 0.005% (*w*/*v*) glucose. Enzymatic pretreatment used 10 µM recombinant SiaBb1 and/or SiaBb2 with 2% (*w*/*v*) 6′-SL or 5% (*w*/*v*) porcine colonic mucin in 10 mM acetate buffer (pH 5.0) for 3 h at 37 °C.	Single- and co-culture growth, recombinant-sialidase treatment, cell enumeration, and sialic-acid-release analysis.	Extracellular release of sialic acid and growth support for *B. breve*.	The *B. bifidum* SiaBb2 sialidase released sialic acid from 6′-SL and supported *B. breve* growth, while SiaBb1 contributed more prominently to mucin utilization. The study demonstrates enzyme-dependent substrate sharing under controlled culture conditions.

**Table 3:** Summary of preclinical and human studies on barrier, immune, and anti-pathogen effects relevant to HMOs and microbial metabolites.

Study	Population or Experimental Model, Design, and Sample Size	Exposure, Dose, and Comparator	Biological Target and Method	Evidence Level and Outcome Type	Main Finding
Gao et al. (2021) [96]	Immature human intestinal epithelial H4 cells and neonatal C57BL/6 mice. Cell findings were derived from three independent experiments where reported. The neonatal-mouse organ-culture experiment was reproduced in two independent experiments with *n* = 6 per experiment.	Butyrate at 20 mM in H4 cells, with or without interleukin-1β at 1 ng/mL. From postnatal day 4, neonatal mice received 10 µL of 100 mM sodium butyrate or PBS by gavage once daily for 3 days and twice daily for the following 4 days; ileum and colon tissues were then incubated ex vivo with or without interleukin-1β. No direct HMO exposure.	Tight-junction, mucin, and inflammatory responses assessed by transcriptional and protein-level assays in H4 cells and neonatal ileum/colon organ cultures.	In vitro + animal—preclinical barrier and inflammatory surrogate.	Butyrate increased selected tight-junction- and mucin-related responses and attenuated selected interleukin-1β-induced inflammatory responses. The study did not demonstrate that the administered butyrate was produced through HMO fermentation and did not establish an effect in human infants.
Laursen et al. (2021) [98]	Danish SKOT I infant cohort *n* = 59 at 9 months; Copenhagen Infant Gut longitudinal cohort *n* = 25 from birth to 6 months; bacterial cultures, monocolonized mice, and ex vivo human CD4+ T-cell and monocyte experiments.	Tryptophan, phenylalanine, and tyrosine as substrates for breastfeeding-associated bifidobacteria; substrate concentrations differed across the bacterial-culture, enzyme-characterization, receptor, and animal experiments and are therefore not summarized as a single dose.	Fecal microbiota/metabolite profiling, aromatic lactate dehydrogenase characterization, bacterial culture, monocolonized mice, receptor assays, and ex vivo immune-cell experiments.	Human observational + in vitro + animal + ex vivo—metabolic and immune surrogate.	Breastfeeding-associated bifidobacteria produced indole-3-lactic, phenyllactic, and 4-hydroxyphenyllactic acids through aromatic lactate dehydrogenase. Infant fecal associations and experimental AhR/HCA3 and immune-cell responses support a mechanistic link, but not prevention of clinical disease.
Ehrlich et al. (2020) [99]	Infant fecal samples *n* = 18 (high-*Bifidobacterium n* = 9; low-*Bifidobacterium n* = 9), *Bifidobacterium infantis* culture, RAW Blue macrophage cells, and Caco-2/HT-29 intestinal epithelial cells.	Indole-3-lactic acid produced by *B. infantis* grown with HMO substrates; dose–response experiments used approximately 0.1–10 mM ILA.	Fecal metabolite comparison; bacterial culture; NF-κB, IL-8, AhR, and Nrf2 pathway assays in macrophage and epithelial-cell models.	Human observational + in vitro—metabolic and inflammatory surrogate.	ILA was enriched in fecal samples with *Bifidobacterium*-dominated microbiota and reduced selected inflammatory responses in vitro, including LPS-induced NF-κB signaling and epithelial IL-8 responses. The human component was observational and did not demonstrate clinical benefit.
Henrick et al. (2021) [100]	Born-immune longitudinal cohort: 208 infants with 858 longitudinal blood samples; fecal metagenomic analyses included 347 samples from 157 infants. The complementary IMPRINT analysis included 60 exclusively breastfed infants receiving *B. longum* subsp. *infantis* EVC001 (*n* = 29) or no supplementation (*n* = 31). Day-21 metagenomics included 60 samples. Fecal cytokine and day-21 metabolomic analyses used randomly selected subsets of 40 infants (*n* = 20 per group); 16S diversity analyses used 80 samples from 40 infants collected on postnatal days 6 and 60. In vitro T-cell experiments used naive CD4+ T cells from one healthy adult donor and pooled fecal water prepared from at least three infants per treatment group.	*B. longum* subsp. *infantis* EVC001 at 1.8 × 10^10^ CFU/day, administered with breast milk from postnatal day 7 through day 28; control infants received breast milk without EVC001. Fecal-water T-cell-polarization experiments used a 1:100 dilution, and indole-3-lactic acid was tested at 1 mM.	Born-immune mass cytometry, plasma-protein profiling, and fecal metagenomics; IMPRINT fecal cytokine, 16S rRNA gene, metagenomic, and metabolomic analyses; and targeted multiomic T-cell-polarization assays.	Human observational + human intervention + in vitro—immune and metabolic surrogate.	In the Born-immune cohort, low bifidobacterial abundance and depletion of HMO-utilization genes were associated with systemic inflammation and altered immune development. In the IMPRINT intervention, EVC001 supplementation was associated with lower intestinal Th2/Th17-related cytokine signals and induction of IFN-β; EVC001-associated indole-3-lactic acid induced galectin-1 during in vitro T-cell polarization. Assay-specific sample sizes should not be assumed to equal either full cohort.
Laucirica et al. (2017) [106]	MA104 African green monkey kidney epithelial cells; in vitro fluorescent-focus infectivity assays; at least three independent experiments per condition.	2′-FL, 3′-SL, 6′-SL, and galacto-oligosaccharides at 2.5 and 5.0 mg/mL; no-oligosaccharide control; timing of addition varied.	Human rotavirus G1P[8] and G2P[4] infectivity measured after glycan treatment at different stages of infection.	In vitro cell-culture infectivity assay—surrogate infectivity endpoint.	All tested glycans reduced infectivity at 5 mg/mL, with virus-strain- and timing-dependent effects. The largest reported reductions were 62% for G1P[8] with 2′-FL added after infection onset and 73% for G2P[4] with a 3′-SL/6′-SL mixture. These cell-culture findings do not establish clinical protection.
Weichert et al. (2016) [107]	Norovirus GII.10 virus-like particles in porcine gastric mucin and A- and B-type saliva surrogate-binding assays; all inhibition assays were performed in triplicate. X-ray crystallography was used to define P-domain-HMO interactions.	2′-fucosyllactose and 3-fucosyllactose were serially diluted from 1 M stocks. Reported IC_50_ values for 2′-FL and 3-FL were 5.5 and 5.6 mM in the porcine gastric mucin assay, 11.2 and 9.7 mM with A-type saliva, and 26.9 and 30.2 mM with B-type saliva. Structural experiments used HMO molar excesses specified in the crystallography protocol.	X-ray crystallography and ELISA-based surrogate HBGA-binding inhibition assays.	In vitro structural/biochemical assay—surrogate binding endpoint.	2′-FL and 3-FL occupied or interfered with norovirus HBGA-binding sites and inhibited GII.10 virus-like-particle binding in surrogate assays. Live-virus infection and clinical norovirus gastroenteritis were not evaluated.
Comstock et al. (2017) [108]	Colostrum-deprived neonatal piglets; randomized dietary study; total *n* = 50 (formula *n* = 16, HMO formula *n* = 17, prebiotic formula *n* = 17); half of each diet group challenged with rotavirus on day 10.	Formula alone versus 4 g HMOs/L (40% 2′-FL, 35% LNnT, 10% 6′-SL, 5% 3′-SL, 10% free sialic acid) versus 4 g prebiotics/L (3.6 g scGOS/L + 0.4 g lcFOS/L).	Peripheral blood, mesenteric lymph node, and ileal Peyer-patch immune populations by flow cytometry; IFN-γ-producing cells by ELISpot at 5 days after infection.	Animal study—preclinical immune surrogate.	HMO-fed piglets had higher circulating NK-cell and selected mesenteric-memory-T-cell populations than formula controls, with altered IFN-γ-producing cells. These immune-cell endpoints were preclinical surrogates and did not demonstrate clinical infection prevention in infants.
Ackerman et al. (2017) [109]	GBS-focused in vitro study using HMO preparations isolated from five donor milk samples. Replicate structure differed by assay; major biofilm experiments were reported across independent experiments with technical replicates, and selected growth assays used biological replicates.	Individual-donor and pooled HMO preparations, generally at approximately 5 mg/mL in the principal growth and biofilm assays.	GBS growth and biofilm formation assessed by culture, MALDI-MS HMO profiling, scanning electron microscopy, and confocal microscopy.	In vitro bacterial culture—mechanistic growth and biofilm endpoints.	HMO preparations from selected donors altered GBS growth and disrupted biofilm architecture in vitro. Activity differed among HMO pools, and the findings support GBS-focused mechanistic and antibiofilm effects only.
Ackerman et al. (2018) [110]	In vitro multi-pathogen study using HMO preparations from 14 new donors; three GBS strains, methicillin-resistant *Staphylococcus aureus* USA300, and *Acinetobacter baumannii* ATCC 19606. Replicate structure was assay specific.	Individual-donor HMO preparations at approximately 5 mg/mL in the primary growth and biofilm screening assays.	Bacterial growth, biofilm formation, and antimicrobial-susceptibility assays.	In vitro bacterial culture—mechanistic antimicrobial and biofilm endpoints.	HMOs showed antimicrobial and/or antibiofilm effects against selected GBS strains, *S. aureus*, and *A. baumannii*. This study expanded beyond the GBS-only focus of the 2017 paper and provides in vitro, not clinical, evidence.
Jarzynka et al. (2021) [111]	Planktonic cultures and mature biofilms of seven pathogenic species. Experiments were independently repeated three times and were performed in technical triplicate where reported.	Total and fractionated HMOs at 5, 10, 20, 50, and 100 mg/mL, with 0 mg/mL controls; selected assays also used 2′-FL, 3′-FL, or lactose comparators.	Planktonic-growth, biofilm-formation, viable-cell, confocal-microscopy, and mature-biofilm-eradication assays.	In vitro bacterial culture—mechanistic growth and biofilm endpoint.	HMO preparations reduced viable cells or disrupted mature biofilms for selected pathogens, with organism-, isolate-, fraction-, and concentration-dependent activity. Effects above 20 mg/mL were not uniformly stronger. The results are restricted to in vitro antimicrobial and antibiofilm activity.
Moore et al. (2023) [112]	Pregnant C57BL/6J mice; adverse-pregnancy-outcome and ascending vaginal GBS-infection models. Adverse-outcome groups: uninfected control, *n* = 5 dams; HMO-only control, *n* = 4; GBS-infected, *n* = 9/group; 2–3 fetal–placental units/dam. Other assays: ≥3 biological replicates. Separate human EpiVaginal and ex vivo gestational-membrane models; adherence assays: 3 independent experiments with 2 technical replicates each.	Pooled HMOs (~5 mg/mL) were administered intravaginally or orally to pregnant mice on E12.5 before vaginal GBS challenge on E13.5 (5 × 10^2^–1 × 10^3^ CFU in the ascending-infection model; 5 × 10^3^–1 × 10^4^ CFU in the adverse-pregnancy-outcome model). Human EpiVaginal and ex vivo gestational-membrane models used 5 mg/mL HMOs; transcriptomic assays used 2.5 mg/mL for 4 h (*n* = 3 cultures/condition).	GBS adherence and biofilm formation in reconstructed EpiVaginal tissue and ex vivo gestational membranes; reproductive-tissue bacterial burden, histopathology, multiplex cytokine responses, pregnancy outcomes, and GBS transcriptomic responses.	Animal + ex vivo human tissue + in vitro transcriptomics—preclinical infection-related surrogate.	Pooled HMOs reduced GBS adherence and biofilm formation in reconstructed vaginal tissue and ex vivo gestational membranes and reduced bacterial burden, selected inflammatory responses, and adverse pregnancy outcomes in preclinical mouse models. These findings are mechanistic and preclinical and do not establish clinical efficacy or safety in pregnant humans or protection of the infant gut.

## Data Availability

No new data were created or analyzed in this study. Data sharing is not applicable to this article.
